# Analytical and Experimental Analysis of a Free Link in Contact with a Granular Medium

**DOI:** 10.1155/2013/808574

**Published:** 2013-11-03

**Authors:** Dan B. Marghitu, Seung Lee

**Affiliations:** Mechanical Engineering Department, Auburn University, 1418 Wiggins Hall, Auburn, AL 36849, USA

## Abstract

In this study, the experimental and the simulation results for a planar free link impacting a granular medium are analyzed. The resistance force of the granular medium on the body from the moment of the impact until the body stops is very important. Horizontal and vertical static resistance forces developed by theoretical and empirical approaches are considered. The penetrating depth of the impacting end of the free link increases with the increase of the initial impacting velocity. We define the stopping time as the time interval from the moment of impact until the vertical velocity of the link end is zero. The stopping time of the end decreases as the initial velocity increases. The faster the end of the link impacts the surface of the granular medium, the sooner it will come to a stop. This phenomenon involves how rapidly a free link strikes the granular medium and how it slows down upon contact.

## 1. Introduction

In industry, the granular materials are the second-most manipulated material (the first one is water) [1]. The most raw materials in nature exist as granules, and many final products are fabricated as granular materials. The study of the granular medium was based on the interaction of the solid particles [2–7] and on the fluid mechanics characteristics [8–10]. The granular materials show similarities and exhibit unusual behaviors compared with solids, liquids, or gases. The contact interactions of a granular material constitute a network of forces at large scale [11–14].

The impact of a solid with a granular material is an important problem because of concomitant contact, collision, and flow phenomena. The penetrating velocity of the element into the granular material influences the major phase state of the granular material. For high-speed impact, the characteristic of the granular material in the vicinity of the body is similar to a fluid, and for slow speed the granular material acts like a solid. For the usual impact cases, the behavior of the granular material exhibits a combined form of solid and fluid characteristics. Earlier studies for high-speed granular material impact were motivated by military applications [15, 16].

For medium- and low-speed penetration, the horizontal resistance force [17], the vertical resistance force [18, 19], the jamming and the fluctuations of the resistance force [20], and the shape effects on the resistance force [21] were studied. The size, the depth, and the form of the crater function of initial impact conditions can be found in [22–27]. A force law model for the granular impacts of dropped spheres represents a new interest in this field [26].

The resistance force models, linear to the depth [25, 28], linear to the velocity [22], and linear to the square of velocity [24], have been studied to explain the motion into the granular materials. Tsimring and Volfson studied the impact cratering by penetration of large projectiles into dry granular medium [23]. They proposed a velocity-dependent drag force and a depth-dependent resistance force. The static resistance force model has been developed for the different motions in [18–21]. Ambroso et al. studied the time dependance for the impacts of rigid sphere [24]. Hou et al. calculated the deceleration of impacting projectiles and concluded that the stopping time is not a linear function of initial impact velocity [25]. The paper of Katsuragi and Durian introduced the resistance force model proposed by [23] for the impact of spheres using a digital CCD camera [26]. Lee and Marghitu extended the theoretical study to the model of a rigid body obliquely impacting the medium [29, 30].

Crassous et al. proposed a model for the propagation of energy due to the impact of a granular projectile on a dense granular medium [31]. A fragment of the kinetic energy of the colliding grain is transferred to the packing and the packing ejects grains. The authors considered a transfer of kinetic energy based on successive binary collisions. Valance and Crassous extended the previous research to a minimal discrete model for the propagation of energy through a 3D granular medium impacted by a particulate projectile [32].

Nguyen and Brogliato simulated the nonlinear wave propagation in granular chains of beads using a multiple impact model. They compare the numerical results with the experimental data [33].

Müller and Pöschel reduce the problem of oblique elastic collisions to two independent parameters and compute the rotation angle as a function of these parameters [34].

The granular materials are ubiquitous, and the impact with a granular medium can take place in various areas such as robotic, human, and animal locomotion, tracked vehicles, and heavy-duty construction equipments. Multilegged kinematic chains cannot avoid the continuous impact with the granular materials. In this study, we focused on modeling, simulation, and experiments of a free kinematic link impacting a granular medium using the resistance force model as the sum of a velocity-dependent drag force and a depth-dependent resistance force. We also analyzed the relation among initial impact velocities, stopping time, and penetrating depth based on the experimental and the simulation results. To the best of our knowledge, this is the first time when a mathematical model is proposed, analyzed, and experimentally verified for a free kinematic link impacting a granular matter.

## 2. Experimental Setup

**Figure 1:**
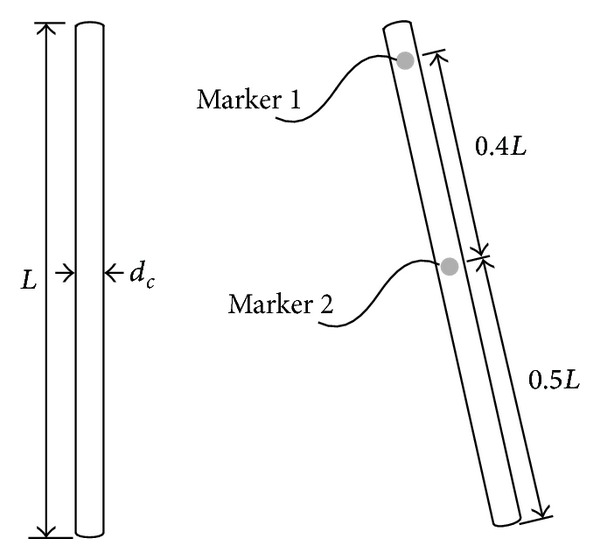
Free link and markers.

**Figure 2:**
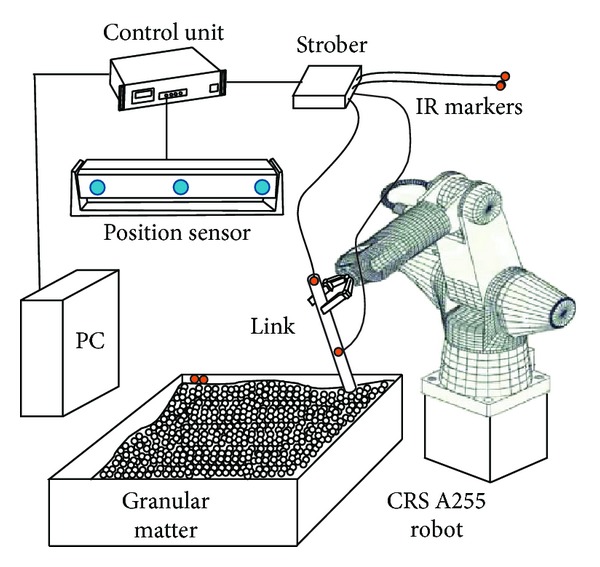
Motion measurement system.

For the free link shown in Figure 1, the following dimensions are given: the length *L* = 0.1524 m and the diameter *d*_*c*_ = 0.00635 m. The density of the link is 7.7 × 10^3^ kg/m^3^. Infrared markers (I.R.) are located on the link at 0.1*L* and 0.5*L* as shown in Figure 1. A motion capture system, Optotrak 3020 (NDI), was used to measure and digitize the position of the impact objects. This system is composed of a position sensor, a control unit, a strober, I.R. markers, and a PC as shown in Figure 2.

The system can measure the position of the markers within the RMS accuracy of 0.1 mm and can track up to 256 markers simultaneously with a sample up to 3500 markers/s [35]. The system does not require a calibration process. The motions of the rigid link are recorded using the two I.R. markers. The sensor captures the position of the I.R. markers attached to the bodies at constant sample rates and measures the 3D position data. The PC is used for operating software, controlling the hardware system, and saving and transforming the measured data. In the experiments, the positions were measured using a cartesian coordinate system at 500 frames/s.

There are many kinds of granular materials: grains such as rice, soils including sand and artificial granules such as fertilizer, glass beads, and ball bearings. For our experiments, we used as granular material “Play sand” (Quikrete 1113-51). The density of the granular medium for the simulations was *ρ*_*g*_ = 2.5 × 10^3^ kg/m^3^. The gravitational acceleration *g* is 9.81 m/s^2^. The impact with a granular medium is related to multibody kinematic chain system such as legged robots, and these systems cannot avoid impacts with outfield granular materials including soil and sand. From these viewpoints, sand is considered more appropriate and beneficial than glass beads or other artificial granular medium. The dimension of impact test box is 0.45 m × 0.32 m × 0.09 m (*W* × *L* × *H*) and the height of the sand in the test bed is 0.075 m.

## 3. Dynamics of the Impact of a Free Link

**Figure 3:**
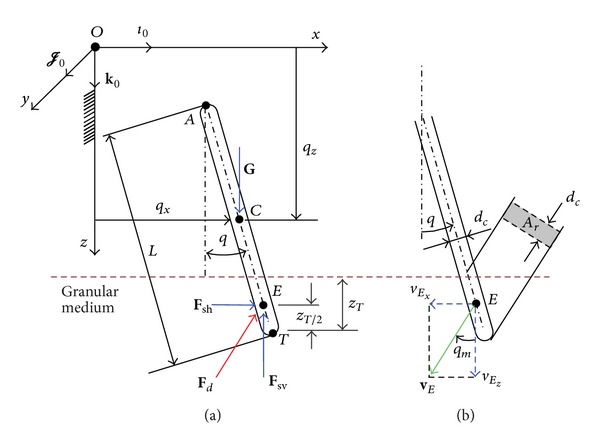
Free link: (a) generalized coordinates and free-body diagram; (b) reference area *A*_*r*_ and moving angle *q*_*m*_.

The free link with the length *L*, the mass *m*_*c*_, and the diameter *d*_*c*_ impact the granular medium as shown in Figure 3(a).

The impact is initiated when the end *T* strikes the surface of the granular material. In order to describe the motion of the rigid free link impacting and penetrating the granular medium, three position coordinates are required for the motion in *xz* plane. Two linear displacements *q*_*x*_ and *q*_*z*_ and one angular position *q* are selected as generalized coordinates of the model. For a flexible free link, the generalized coordinates include the rigid body coordinates and *n* elastic generalized coordinates.

The general equation of motion for the planar kinematic chain can be written in the following form:

(1) $$\begin{array}{c} m_{c}{\ddot{r}}_{C}\cdot {ı}_{0}=\left(F_{s}+F_{d}\right)\cdot {ı}_{0},\\ m_{c}{\ddot{r}}_{C}\cdot k_{0}=\left(G+F_{s}+F_{d}\right)\cdot k_{0},\\ I_{C}\ddot{q}\cdot J_{0}=\left[r_{CE}\times \left(F_{s}+F_{d}\right)\right]\cdot J_{0}, \end{array}$$

where ${\ddot{r}}_{C}$ is the acceleration vector of the mass center of the bar and $\ddot{q}$ is the angular acceleration vector.

The forces acting along the vertical *z*-axis are the gravity force **G**, the vertical static resistance force **F**_sv_, and the vertical component of the dynamic frictional force **F**_*d*_. The forces acting along *x*-axis are the horizontal static resistance force **F**_sh_ and the horizontal component of the dynamic frictional force **F**_*d*_. The gravity force **G** acts at the center of mass, *C*, of the link and the resistance force, **F**_*R*_, including **F**_*s*_ and **F**_*d*_ acting at the point *E*, where point *E* is the centroid of immersed part as shown in Figure 3(a). The resistance force is **F**_*R*_ = **F**_*d*_ + **F**_sh_ + **F**_sv_.

The dynamic frictional force **F**_*d*_ is conceptually the same force as the “drag” used in fluid dynamics. When the external driving forces (including tilting and shaking) exceed the stationary condition, the individual grains loose the stationary state in their contact and the granular material begins to fluidize. From this fluid-like behavior of the granular matter, the resistance force is assumed to be a drag force impeding the body motion [36–40]. The research results using a dilute granular flow condition which is not affected by the static resistance force [36, 38, 40] and the studies at low-speed impact [23, 26] shows the quadratic drag force model is better for the dynamic frictional force **F**_*d*_ than the linear equation model even at relatively low speed. The dynamic frictional force can be modeled as

(2) $$\begin{array}{c} F_{d}=-\frac{v_{E}}{\left|v_{E}\right|}\eta_{d}\rho_{g}A_{r}v_{E}\cdot v_{E}=-v_{E}\eta_{d}\rho_{g}A_{r}\left|v_{E}\right|, \end{array}$$

where **v**_*E*_ = *v*_*E*_*x*__ **ı**_0_ + *v*_*E*_*z*__ **k**_0_ is the velocity vector of the centroid of the immersed part of the link, *η*_*d*_ is an experimental drag coefficient, *ρ*_*g*_ is the density of the granular medium, and *A*_*r*_ is the reference area of the body. For a link in planar motion, as shown in Figure 3(b), the reference area *A*_*r*_ is calculated as

(3) $$\begin{array}{c} A_{r}=d_{c}\frac{z_{T}}{\cos q}\left|\sin\left(q-q_{m}\right)\right|,\\ q_{m}=\tan^{-1}\left(\frac{v_{E_{x}}}{v_{E_{z}}}\right), \end{array}$$

where *q*_*m*_ is a moving angle of the link penetrating the granular medium, as shown in Figure 3(b).

The horizontal static force is defined as an internal resistance force acting on the horizontal direction. When a body penetrates a granular material, it is not easy to separate and to measure this force individually without the effect of dynamic force **F**_*d*_. The experiments for the horizontal static resistance forces were performed at very slow speed such as 0.04–1.4 mm/s [17, 20, 41]. Albert et al. [17] applied a probability approach to model this resistance force.

The horizontal static resistance force of the cylinder including the state of immersion, at any slope, can be generalized as

(4) $$\begin{array}{c} F_{\text{sh}}=-\frac{v_{E_{x}}}{\left|v_{E_{x}}\right|}\eta_{h}g\rho_{g}z_{T}^{2}d_{c}=\left[-\mathrm{sign}\left(v_{E_{x}}\right)\eta_{h}g\rho_{g}z_{T}^{2}d_{c}\right]{ı}_{0}, \end{array}$$

where *η*_*h*_ is an experimental constant [17] and *z*_*T*_ is the depth of the immersed tip as shown in Figure 3(a). Equation (4) shows that the horizontal static resistance force is a function of the granular properties and the depth.

The vertical static force is defined as an internal resistance acting on the vertical axis. Simple models consider this force as a constant [22, 24] or as a linear function of the immersed depth of the body [26, 28]. The vertical static force is also modeled as a nonlinear function of the immersion depth [18, 19]. Experiments show that the granules contact increase exponentially with the external force [13, 42]. The effects of the container bottom boundary increase the nonlinearity of this resistance force [18, 43]. Hill et al. [19] suggested an empirical equation with coefficients calculated from the experimental. The vertical static force for the free link is

(5) Fsv=−vEz|vEz|ηv(zTl)λgρgV=[−sign⁡(vEz)ηv(zTl)λgρgV]k0,

where *V* is the immersed volume of the body and *l* is the lateral dimension. The coefficients *η*_*v*_ and *λ* depend on the shape of the body, the properties of the granular matter, the shape of medium container, and the moving direction such as plunging and withdrawing.

Experimental data show, the inclination of the body has little effect on the vertical static resistance force, but the moving directions change drastically this force [19]. For a cylinder type body, whether the axis is vertical or horizontal, *η*_*v*_ = 10, *λ* = 1.4 for plunging motion and *η*_*v*_ = 0.5, *λ* = 1.7 for withdrawing motion. The lateral dimension *l* is *d*_*c*_, and the immersed volume *V* is calculated with

(6) $$\begin{array}{c} V=\frac{\pi {d_{c}}^{2}}{4}\frac{z_{T}}{\cos q}. \end{array}$$

The resistance force of a cylinder-type link is calculated as

(7) FR=Fd+Fsh+Fsv=[−vExηdρgdczTcos⁡q  ×|  sin(q−tan−1(vExvEz))|vEx2+vEz2  −sign⁡(vEx)ηhgρgzT2  ds]ı0 +[−vEzηdρgdczTcos⁡q   ×|  sin(q−tan−1(vExvEz))|vEx2+vEz2   −sign⁡(vEz)ηv(zTds)λgρgπdc24zTcos⁡q]k0.

The position vector **r**_*CE*_ represents vector from the mass center *C* to the resistance force application point *E*, *m*_*c*_ is the mass of the link, and *I*_*C*_ is the mass moment of inertia of the link with respect to *C*

(8) $$\begin{array}{c} r_{C}=q_{x}{ı}_{0}+q_{z}k_{0},\;\;\ddot{q}=\frac{d^{2}q}{dt^{2}}J_{0}. \end{array}$$

The position **r**_*CE*_ is

(9) $$\begin{array}{c} r_{CE}=L_{CE}\sin q{ı}_{0}+L_{CE}\sin qk_{0}, \end{array}$$

where *L*_*CE*_ is the length between the mass center *C* and the resistance force application point *E*:

(10) $$\begin{array}{c} L_{CE}=\frac{L}{2}-\frac{z_{T}}{2\cos q}. \end{array}$$

The immersed depth of the end *T*, *z*_*T*_, is expressed as

(11) $$\begin{array}{c} z_{T}=r_{C}\cdot k_{0}+\frac{L}{2}\cos q=q_{z}+\frac{L}{2}\cos q. \end{array}$$

The velocity vector **v**_*E*_, the reference area of the penetrating bar *A*_*r*_, and the moving angle *q*_*m*_ are

(12) vE=drCdt+dqdt×rCE=(q˙x+LCEq˙cos⁡q)ı0+(q˙z−LCEq˙sinq)k0,Ar=dczTcos⁡q|sin(q−qm)|,qm=tan−1(vExvEz)=tan−1(q˙x+LCEq˙cos⁡qq˙z−LCEq˙sinq).

The dynamic frictional force **F**_*d*_ has the form

(13) Fd=ηdρgdczTcos⁡q|sin(q−tan−1(q˙x+LCEq˙cos⁡qq˙z−LCEq˙sinq))| ×(q˙x+LCEq˙cos⁡q)2+(q˙z−LCEq˙sinq)2 ×[−(q˙x+LCEq˙cos⁡q)ı0−(q˙z−LCEq˙sinq)k0].

The horizontal and vertical static resistance forces, **F**_sh_ and **F**_vh_, are

(14) $$\begin{array}{c} F_{\text{sh}}=-\mathrm{sign}\left({\dot{q}}_{x}+L_{CE}\dot{q}\cos q\right)\eta_{h}g\rho_{g}z_{T}^{2}d_{c}{ı}_{0},\\ F_{\text{sv}}=-\mathrm{sign}\left({\dot{q}}_{z}-L_{CE}\dot{q}\sin q\right)\eta_{v}{\left(\frac{z_{T}}{d_{c}}\right)}^{\lambda }g\rho_{g}\frac{\pi {d_{c}}^{2}}{4}\frac{z_{T}}{\cos q}k_{0}. \end{array}$$

The resistance force **F**_*R*_, the sum of the dynamic frictional force vector **F**_*d*_ and the static resistance force vector **F**_*s*_, is represented by the sum of (13) and (14) as

(15) FR=Fd+Fsh+Fsv=[−ηdρgdczTcos⁡q  ×|sin(q−tan−1(q˙x+LCEq˙cos⁡qq˙z−LCEq˙sinq))|  ×(q˙x+LCEq˙cos⁡q)2+(q˙z−LCEq˙sinq)2  ×(q˙x+LCEq˙cos⁡q)  −sign⁡(q˙x+LCEq˙cos⁡q)ηhgρgzT2dc]ı0 +[−ηdρgdczTcos⁡q   ×|sin(q−tan−1(q˙x+LCEq˙cos⁡qq˙z−LCEq˙sinq))|   ×(q˙x+LCEq˙cos⁡q)2+(q˙z−LCEq˙sinq)2   ×(q˙z−LCEq˙sinq)   −sign⁡(q˙z−LCEq˙sinq)ηv   ×(zTdc)λgρgπdc24zTcos⁡q]k0.

### 3.1. Results

**Figure 4:**
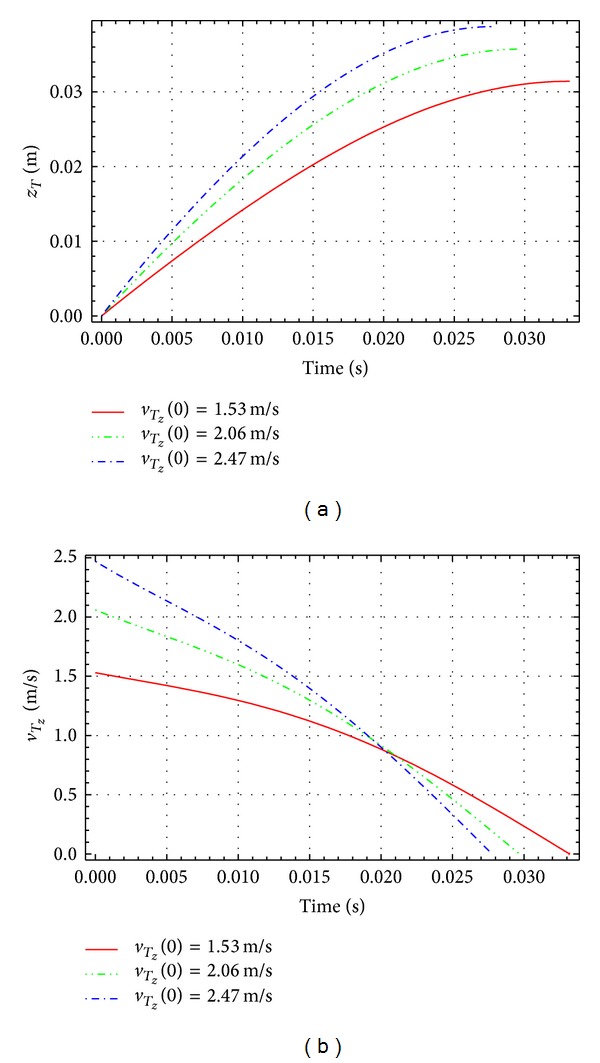
Vertical impact: penetrating depth *z*_*T*_ and vertical velocity *v*_*T*_*z*__.

Figure 4 represents the simulation results of the penetrating depth of the end link, *z*_*T*_, and its vertical velocity *v*_*T*_*z*__, for the vertical impact *q*(0) = 0°. The simulations are performed for different initial impact vertical velocities: ${\dot{q}}_{z}(0)$ = 1.53, 2.06, and 2.47 m/s. We define the stopping time, *t*_*z*_, as the time interval from the moment of impact until the moment when the end *T* stops. At the end of the stopping time the vertical velocity of the link end is *v*_*T*_*z*__ = 0. As shown in Figure 4, the penetrating depth of the end of the link increases with the increase of the initial velocity. Figure 4 shows that the stopping time decreases as the initial impacting velocity increases.

**Figure 5:**
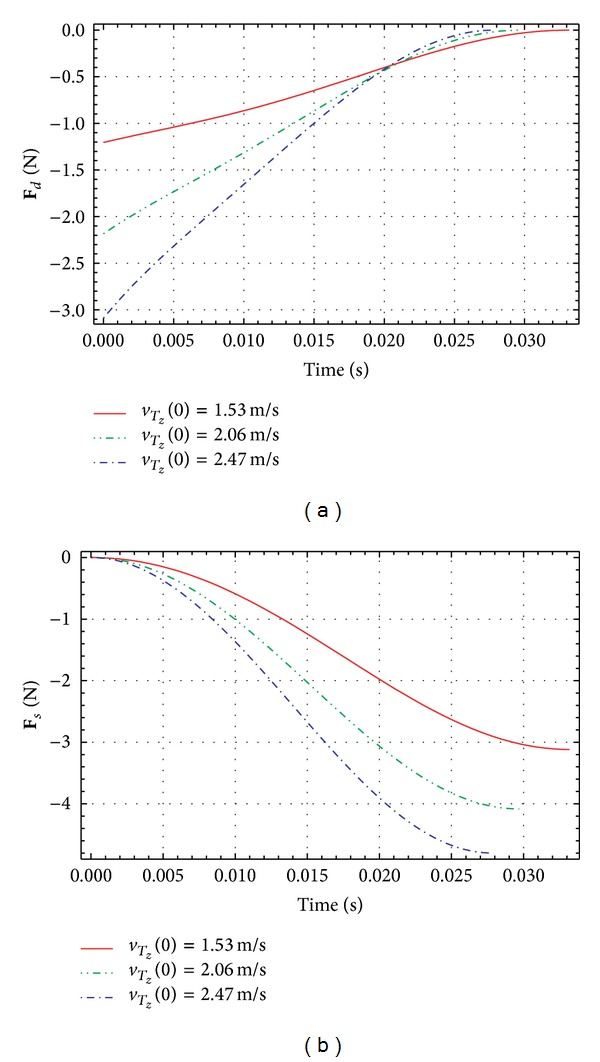
Resistance forces **F**_*d*_ and **F**_*s*_.

Figure 5 depicts the dynamic frictional force and the static force. The dynamic frictional force depending on the velocity acts as a governing resistance force at the beginning of the penetration period. When the penetrating depth increases and the velocity of the end *T* decreases, the static resistance force depending on the immersed depth acts as a governing resistance force.

The simulation results for the stopping time function of initial velocity and initial impact angle are given in Table 1.

**Figure 6:**
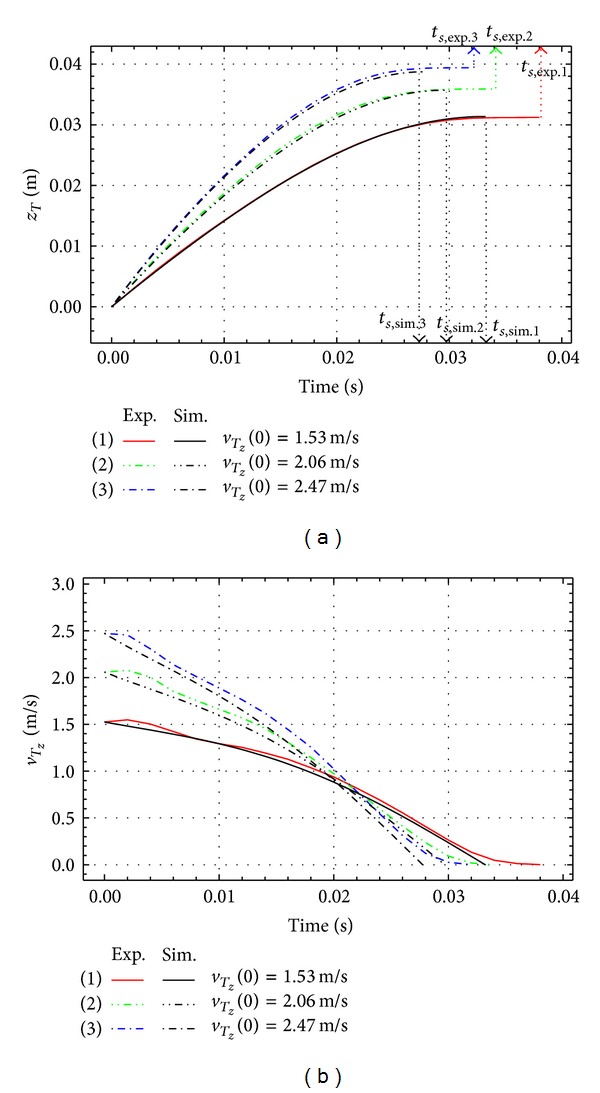
Experimental and simulation results for *q*(0) = 0°.

**Figure 7:**
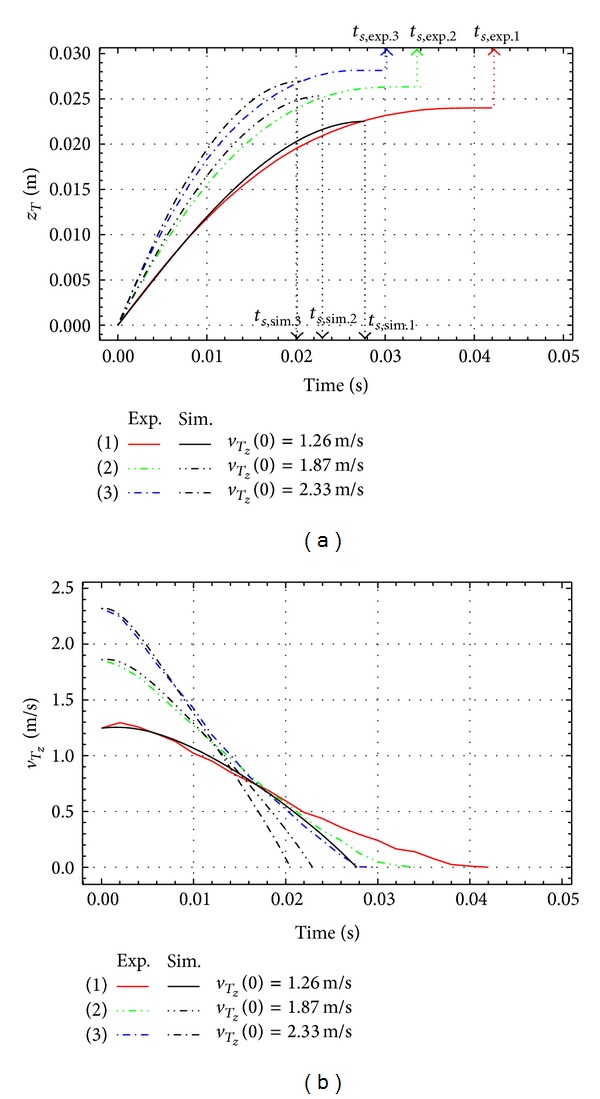
Experimental and simulation results for *q*(0) = 32°.

**Figure 8:**
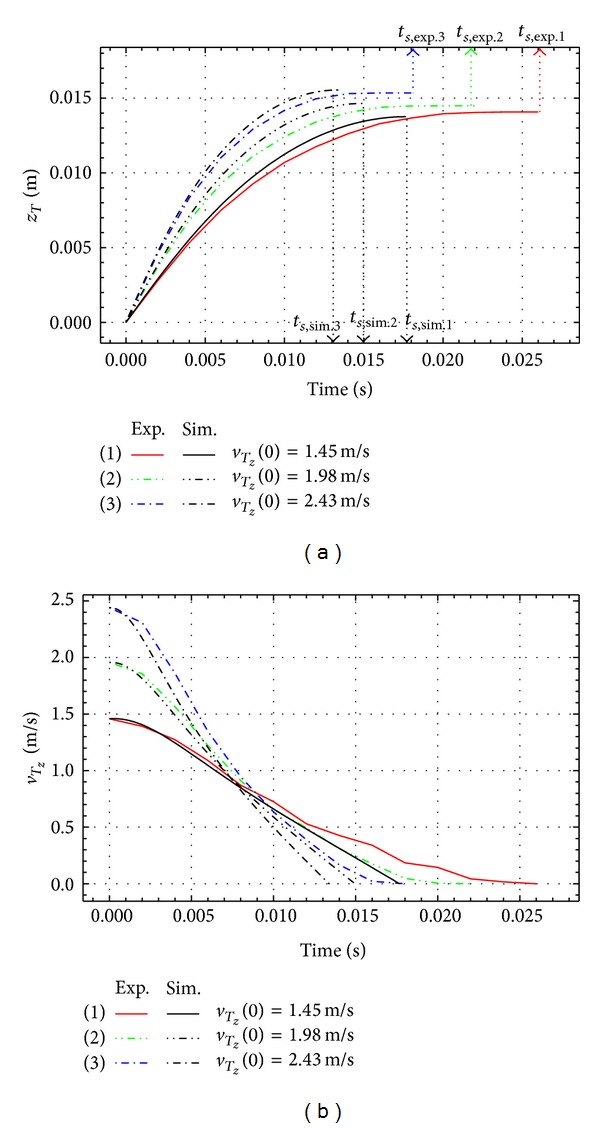
Experimental and simulation results for *q*(0) = 55°.

Figures 6, 7, and 8 represent the experimental and the simulation results for the impact of the free link with different initial impact velocities, ${\dot{q}}_{z}(0)$, and different initial impact angles, *q*(0).

The penetrating depth of *z*_*T*_ and the vertical velocity *v*_*T*_*z*__ are shown in the figures. The initial conditions *q*(0) = 0°, ${\dot{q}}_{z}(0)=1.53$, 2.06, and 2.47 m/s are for Figure 6, *q*(0) = 32°, ${\dot{q}}_{z}(0)=1.26$, 1.87, and 2.33 m/s are for Figure 7, and *q*(0) = 55°, ${\dot{q}}_{z}(0)=1.45$, 1.98, and 2.43 m/s are for Figure 8. Thick lines show the results of experiments and black lines represent the simulation results. The penetrating depth of the link end *T* into the granular matter, *z*_*T*_, is increasing with the initial velocity for all the cases as shown in Figures 6, 7, and 8.

Even there are differences between the simulation and the experimental results, the tendency of the stopping time and the penetrating depth do not change. In this study, a relative error [44] was calculated in order to compare the simulation and the experimental results. The relative error of between the simulation and the experimental results, *γ*, is defined as

(16) $$\begin{array}{c} \gamma =\left|\frac{q_{E}-q_{S}}{q_{E}}\right|\times 100, \end{array}$$

where *q*_*E*_ and *q*_*S*_ mean a position result of experiments and simulations, respectively. In the impact experiments of the link, *q*_*E*_ is considered as the position of the end *T* in the vertical direction, *z*_*T*_.  Table 2 shows the difference rate of the experiments and the simulation.

The stopping time into the granular matter is decreasing when the initial velocity is increasing as most simulation results represented. The characteristics of the stopping time and the penetrating depth do not change.

The vertical velocity *v*_*T*_*z*__ becomes zero faster when the initial vertical impact velocity increases. The increasing of the initial velocity causes the stopping time into the granular medium to decrease. The faster the end of the link impacts the surface of the granular medium, the sooner it will come to a stop. This is an interesting phenomenon involving how rapidly a body vertically strikes the granular medium slowing down upon contact. The results can be explained by the reaction of the granular medium on the free link that can be decomposed into the sum of velocity-dependent force and depth-dependent force [26].

## 4. Conclusions

The experimental and the simulation results for the oblique impact of a free link impacting with a granular medium are analyzed. The resistance forces acting during the penetration of the free link into the granular matter is modeled as the sum of the static force represented by a depth-dependent friction force as well as the dynamic frictional force which is a velocity-dependent drag force. The penetrating depth of the impacting end of the free link increases with the increase of the initial impact velocity. The stopping time of the impacting end in vertical direction decreases as the initial impacting velocity increases. The faster the end of the link impacts the surface of the granular medium, the sooner it will come to a stop.

This research provides a new strategy in the theory of general impacting bodies with granular materials. This study is very useful in the design of impacting systems such as walking machines, variable geometry wheeled and tracked vehicles, active cord mechanisms, and robot manipulators. The results obtained are significant in the areas of mining, military transport, planetary exploration, construction work on land and under water, and study of locomotion.

## Figures and Tables

**Table 1:** Stopping time for the free link.

*q*(0) (°)	*v*_*T*_*z*__(0) (m/s)	*t*_*z*_ (s)
0	1.53	0.033
	2.06	0.029
	2.47	0.027

32	1.26	0.027
	1.87	0.022
	2.33	0.020

55	1.45	0.017
	1.98	0.015
	2.43	0.013

**Table 2:** Relative error between the experimental and the simulation results of the free link.

Impact angle (°)	*v*_*T*_*z*__(0) (m/s)	γ (%)
0	1.53	0.52
	2.06	0.54
	2.47	1.68

32	1.26	6.19
	1.87	3.85
	2.33	4.28

55	1.45	2.25
	1.98	1.10
	2.43	1.32
